# A Survey of Current Practices and Preferences for Internal Fixation of Trochanteric Fractures of the Femur in Brazil

**DOI:** 10.7759/cureus.2286

**Published:** 2018-03-07

**Authors:** Vincenzo Giordano, Denner N Ribeiro, Rafael G Tinoco, Thiago A Alvim, Marcos Giordano, Anderson Freitas, Hilton A Koch

**Affiliations:** 1 Department of Orthopaedics, Hospital Municipal Miguel Couto (OST-HMMC); 2 Trauma and Orthopedics Department, Galeão Air Force Hospital; 3 Hip Department, Hospital of Orthopedic and Specialized Medicine (home); 4 Department of Radiology, Federal University of Rio De Janeiro

**Keywords:** femur, fracture fixation, fracture fixation - internal, orthopedic fixation devices, hip fractures

## Abstract

Objective

The primary aim of this study was to survey current practices and preferences behind internal fixation of trochanteric femoral fractures among Brazilian orthopedic surgeons. The secondary aim was to identify the main reason for these preferences.

Methods

A survey containing 20 images of trochanteric fractures of the femur was presented to a group of 62 orthopedists, all members of the Brazilian Society of Orthopedics and Traumatology (SBOT). The first part of the questionnaire was created to identify the surgeons’ degree of professional experience, type of practice, and areas of greatest interest and performance within the specialty. The second part of the questionnaire contained options for fixating different trochanteric fracture patterns in the femur for participants to choose, along with the main reason for their decision. Statistical analysis was descriptive and profiled the surgeons’ major area of interest, treatment option, and the main reason for their therapeutic decision.

Results

Of the 62 orthopedists who participated in the study, 10 (16.0%) stated that their area of greatest interest was orthopedic trauma and 52 (83.9%) reported greater interest in another area of the specialty; these two groups were classified as the Trauma Group and Orthopedics Group, respectively. To treat AO 31A1 type fractures, the trauma group selected the sliding hip screw (SHS) in 66.7% of cases, while the orthopedics group chose the SHS in 65.8% of cases. For 31A2 type fractures, the trauma group chose the intramedullary (IM) nail in 64.0% of the cases, while the orthopedics group chose the IM nail in 76.7% of the cases. For 31A3 type fractures, the trauma group opted for the IM nail in 70.0% of the cases, while the orthopedics group selected the IM nail in 88.0% of the cases. The two most important factors in implant selection for the three types of fracture were fracture pattern and implant availability.

Conclusion

The sliding hip screw is preferred by most Brazilian orthopedic surgeons for fixation of 31A1 type trochanteric femoral fractures. For 31A2 and 31A3 type fractures, the IM nail is preferred.

## Introduction

Treatment of trochanteric fracture of the femur seems well-defined in the literature. In 1980, Kaufer identified five factors related to the final outcome of surgical treatment of this lesion, including implant choice [1]. The ideal implant should consider the morphology of the fracture, degree of instability, the patient's functional independence, and the cost of surgical materials, among other factors [2,3]. After changes that resulted from recognition of the importance of soft tissue to bone biology, the search for minimally invasive techniques has led to different options for fixation and techniques to treat the proximal extremity of the femur [4].

Previous studies indicated that the sliding hip screw (SHS) was preferred by most authors [4,5]. This implant provides stability for the fracture by dividing the load between the bone and the implant, allowing a controlled collapse of the proximal fragment over the distal fragment [5,6]. But use of the SHS is questioned for more unstable fracture patterns, such as those involving reverse obliquity, extension of the fracture into the subtrochanteric region, or involvement of the lateral wall of the femur [2,7,8]. In these cases, intramedullary (IM) implants are generally recommended for their more favorable biomechanical characteristics, which reduce the risk of fixation failure as well as the rate of complications [2]. Even so, debate continues over which internal fixation method is ideal for treating most trochanteric fractures.

Several studies have failed to show that IM implants are superior to the SHS, since both are associated with similar results in most trochanteric fracture patterns [9-11]. Nevertheless, the use of IM implants has grown in recent years, even in stable trochanteric fractures, mainly among younger American orthopedic surgeons. In Brazil, however, little is known about which implants orthopedic surgeons prefer for internal fixation of trochanteric femur fracture, whether intra- or extramedullary. Consequently, the primary objective of this study was to survey current practices and preferences in Brazil for internal fixation of trochanteric fracture of the femur. A secondary objective was to identify the main reason for these decisions.

## Materials and methods

A two-part questionnaire was developed. The first section of the questionnaire was designed to identify participants’ degree of professional experience, practice type, and area of greatest interest and activity within the specialty. The second part of the questionnaire presented fixation options for different patterns of trochanteric femur fracture; participants selected the option they considered best and provided the main reason for their decision. The questionnaire used is included with this study (Appendix 1).

We used 20 X-ray images showing trochanteric femur fractures in the anteroposterior (AP) and profile views of the fractured hip, and a panoramic AP view of the pelvis. The fractures were previously classified by two active members of the Brazilian Society of Orthopedic Traumatology (SBOT) using the AO classification system, and there was no disagreement. The images used depicted five 31A1 fractures, 10 31A2 fractures, and five 31A3 fractures. The choice of fracture type was intended to cover stable (31A1) and unstable fracture patterns (31A2 and 31A3). The questionnaire containing 20 images was presented to a group of 62 orthopedic physicians, all members of the Brazilian Society of Orthopedics and Traumatology (SBOT).

The statistical analysis was essentially descriptive and created a profile according to different aspects: surgeon’s area of greatest interest, treatment option, and main reason for the therapeutic decision. The summarized data are presented as tables and expressed by frequency and percentage. The statistical analysis was conducted using SAS software version 6.11 (SAS Institute Inc., Cary, North Carolina).

## Results

Part one: respondent profile

Of the 62 orthopedic physicians who participated in the study, only 10 (16.0%) reported their area of greatest interest as trauma, with the rest (52 respondents - 83.9%) stating interest in another specialty area. We separated these respondents into two groups, the Trauma Group and the Orthopedics Group, respectively.

Most participants reported performing up to three surgeries to repair fractures of the proximal end of the femur per month (34 respondents - 54.8%), while 21 (33.9%) reported performing four to six surgeries per month, and seven (11.3%) more than six surgeries per month.

With regard to professional experience in this specialty, the majority of respondents reported up to five years of orthopedic training (45 respondents - 72.6%), 13 (21%) between five and 10 years, and four (6.5%) more than 10 years of training.

The data collected in the first part of the questionnaire are presented in Table 1.

**Table 1:** Surgeon characteristics

Questions	N	%
What is your area of interest/activity?		
Trauma Group	10	16.1
Orthopedics Group	52	83.9
How many fractures of the proximal end of the femur do you treat surgically per month, on average?		
Up to three	34	54.8
Four to six	21	33.9
More than six	7	11.3
How many years of professional experience do you have in orthopedics?		
Less than five	45	72.6
Five to 10	13	21.0
More than 10	4	6.5

Part two: fracture treatment preference

In choosing internal fixation implants to treat each of the 20 cases of trochanteric fracture, both groups had a similar preference for the SHS in treating 31A1 (stable) fractures (66.7% (28) among the trauma group, 65.8% (171) in the orthopedics group). To treat 31A2 (unstable) fractures, participants in the orthopedics group chose IM nails more frequently than the trauma group (76.7% (396) of cases versus 64.0% (55), respectively). For 31A3 (unstable) fractures, the orthopedics group selected IM nails even more often than the trauma group (88.0% (227) of cases versus 70.0% (28) of cases, respectively). The data can be seen in Table 2.

**Table 2:** Distribution of treatment by fracture type and surgeon specialty

Fracture	Treatment	Total N %	Trauma Group N %	Ortho Group N %
31A1	SHS	199 65.9	28 66.7	171 65.8
	IM nail	103 34.1	14 33.3	89 34.2
31A2	SHS	151 25.1	31 36.0	120 23.3
	IM nail	451 74.9	55 64.0	396 76.7
31A3	SHS	43 14.4	12 30.0	31 12.0
	IM nail	255 85.6	28 70.0	227 88.0
Total	SHS	393 32.7	71 42.3	322 31.1
	IM nail	809 67.3	97 57.7	712 68.9

In the trauma group, the primary reason for choosing the SHS to treat 31A1 fractures was fracture pattern, followed by implant availability (64.3% (18) versus 21.0% (six), respectively). In the orthopedics group, the vast majority of surgeons justified their preference for the SHS to treat 31A1 fractures as fracture pattern, followed by implant availability (81.9% (140) versus 2.3% (four), respectively). For this same type of fracture, surgeons in the trauma group justified selecting IM nails because of the fracture line in three cases (21.4%), and medial fragmentation, involvement of the lateral wall, and possibility of intraoperative fracture of the lateral wall in two cases (14.3%) each. In the orthopedics group, the main reason for choosing IM nails was the fracture line in 24 cases (27.0%), involvement of the lateral wall in 16 cases (18.0%), and medial fragmentation in 14 cases (15.7%). These data are presented in Tables 3-4.

To treat 31A2 fractures, the trauma group justified the use of IM nails because of medial fragmentation in 19 cases (34.5%), and the possibility of intraoperative fracture of the lateral wall in 11 cases (20.0%). In the orthopedics group, the main reason for choosing IM nails was medial fragmentation in 205 cases (51.8%), followed by involvement of the lateral wall in 76 cases (19.2%). In the trauma group, the main reason for choosing the SHS was medial fragmentation in 13 cases (41.9%), and fracture pattern in 11 cases (35.5%). In the orthopedics group, the main reason for choosing the SHS was the fracture pattern in 66 cases (55.0%) and implant availability in 12 cases (10.0%). These data are presented in Tables 3-4.

In the trauma group, the main reason for choosing the IM nail to treat 31A3 fractures was the fracture line in 16 cases (57.1%), and involvement of the lateral wall in seven cases (25.0%). In the orthopedics group, the main reason for selecting IM nails was medial fragmentation in 75 cases (33.0%), fracture line in 72 cases (31.7%), and involvement of the lateral wall in 44 cases (19.4%). In the trauma group, the main reason for choosing the SHS was equally divided between fracture pattern and implant availability, with six cases (50.0%) each. In the orthopedics group, the main reason for choosing the SHS was the fracture pattern in 18 cases (58.1%) and implant availability in 12 cases (12.9%). These data are presented in Tables 3-4.

**Table 3:** Reason for implant choice in trauma group sample according to total number and type of treatment PMF: posteromedial fragmentation LWI: lateral wall involvement PLWF: possibility of lateral wall fracture FP: fracture pattern IA: implant availability LBL + LMD: less blood loss + less muscle damage OFL: orientation of the fracture line

Fracture	Code	Reason	Total			SHS			IM nail	
			n	%		n	%		N	%
31A1	1	PMF	2	4.8		0	0.0		2	14.3
	2	LWI	2	4.8		0	0.0		2	14.3
	3	PLWF	3	7.1		1	3.6		2	14.3
	4	FP	18	42.9		18	64.3		0	0.0
	5	IA	6	14.3		6	21.4		0	0.0
	6	LBL+LMD	0	0.0		0	0.0		0	0.0
	7	OLF	3	7.1		0	0.0		3	21.4
	8	Other	8	19.0		3	10.7		5	35.7
31A2	1	PMF	19	22.1		0	0.0		19	34.5
	2	LWI	7	8.1		0	0.0		7	12.7
	3	PLWF	11	12.8		0	0.0		11	20.0
	4	FP	11	12.8		11	35.5		0	0.0
	5	IA	15	17.4		13	41.9		2	3.6
	6	LBL+LMD	5	5.8		0	0.0		5	9.1
	7	OLF	7	8.1		0	0.0		7	12.7
	8	Other	11	12.8		7	22.6		4	7.3
31A3	1	PMF	3	7.5		0	0.0		3	10.7
	2	LWI	7	17.5		0	0.0		7	25.0
	3	PLWF	0	0.0		0	0.0		0	0.0
	4	FP	6	15.0		6	50.0		0	0.0
	5	IA	6	15.0		6	50.0		0	0.0
	6	LBL+LMD	0	0.0		0	0.0		0	0.0
	7	OLF	16	40.0		0	0.0		16	57.1
	8	Other	2	5.0		0	0.0		2	7.1

**Table 4:** Trauma group sample: distribution of reasons for selecting implant according to total and type of treatment PMF: posteromedial fragmentation LWI: lateral wall involvement PLWF: possibility of lateral wall fracture FP: fracture pattern IA: implant availability LBL + LMD: less blood loss + less muscle damage OFL: orientation of the fracture line

Fracture	Code	Reason	Total			SHS			IM nail	
			N	%		N	%		N	%
A1 Fracture	1	PMF	15	5.8		1	0.6		14	15.7
	2	LWI	19	7.3		3	1.8		16	18.0
	3	PLWF	12	4.6		2	1.2		10	11.2
	4	FP	143	55.0		140	81.9		3	3.4
	5	IA	8	3.1		4	2.3		4	4.5
	6	LBL+LMD	10	3.8		2	1.2		8	9.0
	7	OLF	24	9.2		0	0.0		24	27.0
	8	Other	29	11.2		19	11.1		10	11.2
A2 Fracture	1	PMF	208	40.3		3	2.5		205	51.8
	2	LWI	82	15.9		6	5.0		76	19.2
	3	PLWF	41	7.9		2	1.7		39	9.8
	4	FP	73	14.1		66	55.0		7	1.8
	5	IA	23	4.5		12	10.0		11	2.8
	6	LBL+LMD	13	2.5		2	1.7		11	2.8
	7	OLF	18	3.5		1	0.8		17	4.3
	8	Other	58	11.2		28	23.3		30	7.6
A3 Fracture	1	PMF	75	29.1		0	0.0		75	33.0
	2	LWI	45	17.4		1	3.2		44	19.4
	3	PLWF	8	3.1		2	6.5		6	2.6
	4	FP	18	7.0		18	58.1		0	0.0
	5	IA	9	3.5		4	12.9		5	2.2
	6	LBL+LMD	8	3.1		1	3.2		7	3.1
	7	OLF	73	28.3		1	3.2		72	31.7
	8	Other	22	8.5		4	12.9		18	7.9

## Discussion

The surgeons who participated in this study preferred the SHS for 31A1 fractures and the IM nail for 31A2 and 31A3 fractures, regardless of whether their major interest was trauma or another area. This probably reflects the preference of most Brazilian orthopedists who treat trochanteric fractures of the femur in their everyday practice. There are clear advantages and disadvantages for each method, as well as recommendations depending on the fracture pattern. In 2014, a volunteer work group comprising members of the American Academy of Orthopedic Surgeons (AAOS) developed Clinical Practice Guidelines based on a systematic review of current scientific and clinical information and current approaches to treating trochanteric fractures of the femur [12]. Even so, implant choice still greatly depends on the surgeon's preference and training, as well as the availability of materials for internal fixation.

Specifically, in the case of trochanteric fractures, other factors were seen to be relevant in implant selection, such as fragmentation of the posterior-medial region (involvement of the small trochanter) and primary involvement or risk of intraoperative fracture of the lateral wall of the proximal end of the femur. Recognition of the morphological characteristics that could lead to instability in trochanteric fractures after reduction, such as reverse obliquity of fracture trace and subtrochanteric extension, is fundamental in selecting the implant and reducing the risk of fixation failure [2,7,8]. Some moderate evidence supports the use of intramedullary devices to treat patients with unstable trochanteric fractures [12]. In contrast, in patients with stable trochanteric fracture, moderate evidence supports the use of the SHS [12].

In this current study, fracture pattern was the most important factor in choosing the SHS to treat 31A1 fractures among physicians in the trauma group (64.3%) as well as the orthopedics group (81.9%). Approximately one-fifth (21.4%) of the trauma group responded that implant availability was the main reason they selected the SHS. Although it cannot be definitively proven, this finding permits the assumption that despite the morphological stability of this fracture pattern, this group would prefer IM devices if these were available. This behavior can be explained (at least partly) through more specific knowledge of the biomechanical advantages of intramedullary devices in this region, such as better load sharing because of its central location in the load axis and less shortening of the femur neck, which in turn has less of an effect on hip offset [10,13]. Interestingly, cost of the implant was not mentioned as a priority factor in the decision-making process of what type of fixation is preferred for this type of fracture, although the economic aspect is extremely relevant in a country like Brazil.

In 31A2 fractures, fracture pattern was an important factor in selecting the IM nail for both groups (35.5% in the trauma group and 55.0% in the orthopedics group), although implant availability was the most important factor for decision-making in the trauma group (41.9%). This corroborates the authors’ hypothesis that more specific knowledge of intramedullary devices may have influenced implant preference. For many physicians, the presence of fractures in the small trochanter region is automatically interpreted as a marker of instability, indicating the use of intramedullary devices [2]. Saudan et al. called attention to the enormous variability of 31A2 fracture presentations, which generally involve different degrees of fragmentation of the posteromedial wall at the proximal end of the femur and a varying spectrum of instability [11]. In most cases, there is little involvement of the small trochanter region, and this does not interfere with the controlled compression which the SHS provides on the primary fracture line [2]. In fact, several studies have shown similar results for the SHS and intramedullary fixation devices in treating this type of fracture [11,13-20]. In these cases, intramedullary nails only have a lower failure rate when there is a great deal of posterior-medial fragmentation with loss of support from the calcar [2]. The difficulty involved in radiographic interpretation of the degree of involvement in the small trochanter region and the thickness of the lateral wall led these authors to conduct computed tomographic studies on patients with 31A2 type fractures.

In 31A3 fractures, fracture pattern again was an important factor for selecting IM nails in both groups (50.0% in the trauma group and 58.1% in the orthopedics group). For half (50.0%) of the participants in the trauma group, implant availability was the most significant factor in decision-making. In this type of fracture, strong evidence supports the use of IM nails [12]. The unfavorable biomechanics of this injury, mainly due to loss of the medial and lateral walls, rules out fixation with the SHS since the direction of a controlled collapse (which usually occurs in trochanteric fracture) is not perpendicular to the primary fracture line and the lateral cortex is not present to resist this collapse [21].

This study has a number of limitations. The first is the small number of participants, which may question whether our results can be extrapolated to indicate the preferences among orthopedic physicians throughout the country. However, everyone who was invited to participate completed the questionnaire, indicating widespread interest as well as the participants’ degree of understanding on this topic. Another limitation was the lack of information about where the participants work, namely public or private hospitals. Since medicine in Brazil varies between the public and private health networks in terms of access to implantable surgical materials, lack of implant options may have led some surgeons to select extramedullary implants in situations where they would normally prefer to use intramedullary ones. In fact, implant availability was among the most important factors in selecting internal fixation. Nevertheless, our results contrast with global trends preferring the use of IM nails to treat 31A2 and 31A3 fractures, showing that while this information is relevant, it did not impact participants’ choices.

## Conclusions

The sliding hip screw is the implant of choice among most Brazilian orthopedists for fixation of 31A1 trochanteric fractures of the femur. For 31A2 and 31A3 fractures, the intramedullary nail is preferred among this group. The two most important factors in selecting implants to treat these three fracture types were fracture pattern and implant availability.

## Appendices

Questionnaire used to assess fixation preference for femoral trochanter fractures among Brazilian orthopedists

1. Based on the X-rays, how would you treat this fracture?

□                Sliding hip screw

□                Proximal femoral nail

2. What is the main reason for your decision?

□                Posteromedial comminution

□                Involvement of the lateral wall

□                Possible intraoperative fracture of the lateral wall

□                Stable fracture pattern

□                Implant availability

□                Less blood loss and muscle injury

□                Reverse fracture line

□                Other
